# Supplementary material for: Determinants of condom use during last sexual intercourse among male college youth of Kaski, Nepal: A cross-sectional survey
Source: PLoS One. 2021 Dec 30;16(12):e0261501. doi: 10.1371/journal.pone.0261501 (PMC8717988; doi:10.1371/journal.pone.0261501)
Supplement: S1 File — (DOCX) [file pone.0261501.s002.docx]

**QUESTIONNAIRE**

**SECTION A: SOCIO DEMOGRAPHIC INFORMATION**

| A1 | Age | ………years |
| --- | --- | --- |
| A2 | Are you permanent residence of Pokhara metropolitan? | i. Yes  ii. No |
| A3 | Marital status | i. Married  ii. Unmarried |
| A4 | Religion | i.Hindu  ii.Buddhist  iii.Muslim  iv.Christain  v.Others (Specify it)……………. |
| A6 | With whom you are currently living with? | i.Family  ii. Friend’s  iii. Relatives  iv Others (Specify it)……………. |

**SECTION B : KNOWLEDGE ABOUT CONDOM AND ATTITUDE TOWARDS CONDOM**

| S.N. | Items | Agree | Don’t know/Not sure | Disagree |
| --- | --- | --- | --- | --- |
| D1 | Condoms are an effective method of preventing pregnancy |  |  |  |
| D2 | Condoms can be used more than once |  |  |  |
| D3 | A girl can suggest to her boyfriend that he use a condom |  |  |  |
| D4 | A boy can suggest to his girlfriend that he use a condom |  |  |  |
| D5 | Condoms are an effective way of protecting against HIV/AIDS |  |  |  |
| D6 | Condoms are suitable for casual relationships |  |  |  |
| D7 | Condoms are suitable for steady, loving relationships. |  |  |  |
| D8 | It would be too embarrassing for someone like me to buy or obtain condoms |  |  |  |
| D9 | If a girl suggested using condoms to her partner, it would mean that she didn't trust him |  |  |  |
| D10 | Condoms reduce sexual pleasure |  |  |  |
| D11 | Condoms can slip off the man and disappear inside the woman's body |  |  |  |
| D12 | If unmarried couples want to have sexual intercourse before marriage, they should use condoms |  |  |  |
| D13 | Condoms are an effective way of protecting against sexually transmitted diseases |  |  |  |

**SECTION C: SEXUAL BEHAVIOR**

| **S.N.** | **Items** | **Responses** | **Remarks** |
| --- | --- | --- | --- |
| E1 | Do you have an unmarried friend who has experienced sexual intercourse? | i. Yes  ii.No |  |
| E2 | Have you ever been involved in the following activities ?(Tick for the options you were involved ) | i.Masturbation  ii.Kissing  iii.Vaginal intercourse  iv.Anal sex | Go to E3 if your response is iii or iv or iii and iv  If your response doesnot include iii or iv or iii and iv then thank you for participating in this survey |
| E3 | What was your age when you had your first sexual intercourse? | i.Age ……years  ii.Don’t remember |  |
| E4 | Did you or your partner used any contraceptive during your first sexual intercourse? | i.Yes  ii.No | Go to E5 if your response is yes if your response is No go to E6 |
| E5. | Which contraceptive did you or your sexual partner used during the first sexual intercourse? | i.Female condom  ii.Male condom  iii.Contraceptive pills  iv.Contraceptive injection  v.Emergency contraceptive pills |  |
| E6 | Did you or your partner used any alternative measures as withdrawal, sexual intercourse during safe period on your first sexual intercourse? | i.Yes  ii.No | Go to E7 if your response is yes if your response is No go to E8 |
| E7 | Which alternative measures did you or your partner used during your first sexual intercourse? | i. Withdrawal  ii.Safe period |  |
| E8 | How many sexual partners have you established sexual relations till now | ……….. |  |
| E9 | When did your last sexual intercourse occurred ? | i.………….Days ago  ii.………… Months ago |  |
| E10 | What is your relationship with the sexual partner with whom your latest sexual intercourse occurred ? | i.Wife  ii.Girlfriend  iii.Casual friend  iv.Client  v.If others please specify it ……………… |  |
| E11 | Did you or your partner used any contraceptive devices during your last sexual intercourse? | i.Yes  ii.No | Go to E12 if your response is yes if your response is No go to E13 |
| E12 | Which contraceptive did you or your sexual partner used during the last sexual intercourse? | i.Female condom  ii.Male condom  iii.Contraceptive pills  iv.Contraceptive injection  v.Emergency contraceptive pills |  |
| E13 | Did you or your partner used any alternative measures as : withdrawal, sexual intercourse during safe period during your last sexual intercourse? | i.Yes  ii.No | Go to E14 if your response is yes if your response is No thank you for participating |
| E15 | Which alternative measures did or your partner used during your last sexual intercourse? | i.Withdrawal  ii.Safe period |  |

***END***

***THANK YOU FOR PARTICIPATING IN THIS STUDY***

**Questionnaire in Nepali form**

**भाग १M सामाजिक जनसाङ्खिक विवरण**

| क्र.स. | प्रश्न | प्रतिक्रियाहरु |
| --- | --- | --- |
| A1 | उमेर | ………… वर्ष |
| A2 | के तपाई पोखराको स्थायी वासिन्दा हो? | क.हो ख.होइन |
| A3 | वैवाहिक अवस्था | क.अविवाहित ख.विवाहित |
| A4 | धर्म | क. हिन्दु ख. बुद्धिजम ग. मुस्लिम घ. क्रिस्यियन  ङ. अन्य ........ |
| A5 | हालै तपाईकोसँग बस्नुहुन्छ? | क.परिवार ख.आफन्त ग.साथी घ.अन्य ........ |
| A6 | आम्दानीको श्रोत | क.जागिर ख.व्यापार  ग.आफ्नै आम्दानीको श्रोत छैन |

**भाग २ :कण्डम सम्बन्धि ज्ञान र कण्डम प्रतिको दृष्टिकोण**

यदी तपाइ दिइएको भनाइहरुप्रति सहमत जनाउनु हुन्छ भने त्यसै अनुसार दिइएको छनोटमा चिन्ह(✔ ) लगाउनु होला।यदी थाहा छैन भने सोही छनोटमा चिन्ह लगाउनु होला।

| क्र.स. | भनाइहरु | सहमत छु | थाहा छैन | सहमत छैन |
| --- | --- | --- | --- | --- |
| D1 | कन्डमको प्रयोगले योजना बिहिन गर्वबती हुनबाट बचाउछ |  |  |  |
| D2 | एकपटक प्रयोग गरेको कण्डम अर्को पटकपनि प्रयोग गर्न मिल्छ |  |  |  |
| D3 | महिलाले आफ्नो पुरुष यौन साथीलाई कण्डम प्रयोग गर्न प्रस्ताब राख्न सक्छिन् |  |  |  |
| D4 | एक पुरुषले आफ्नो महिला यौन साथीलाई कण्डम प्रयोग गर्न प्रस्ताब राख्न सक्छन |  |  |  |
| D5 | कन्डमको प्रयोगले एच.आइ.भी. लाग्न बाट बचाउछ |  |  |  |
| D6 | आकष्मिक सेक्सको लागि कण्डमकोप्रयोग जरुरि छ |  |  |  |
| D7 | एक स्थिर प्रेम सम्बन्धको लागि कन्डमको प्रयोग जरुरि छ |  |  |  |
| D8 | म जस्तोव्यक्तीलाइकण्डम किन्न वा लिन सरम लाग्छ |  |  |  |
| D9 | एक महिलाले आफ्नो पुरुष यौन साथीलाई कण्डम प्रयोग गर्न प्रस्ताब राखिन भने उनले आफ्नो साथीलाई बिश्वास गर्दिनन |  |  |  |
| D10 | कन्डमको प्रयोग गर्नाले यौन सन्तुस्टी घटाउछ |  |  |  |
| D11 | कन्डम चिप्लिएर महिलाको ज्यान भित्र हराउन सक्छ |  |  |  |
| D12 | अबिबाहित प्रेमी प्रेमिकाले बिबाह अगाडी यौन सम्पर्क राख्नु परेमा कन्डमको प्रयोग गर्नुपर्छ |  |  |  |
| D13 | कन्डमको प्रयोगले यौन रोगबाट बचाउछ |  |  |  |

**भाग ३: यौनव्यवहार**

| qm=;+= | प्रश्न | प्रतिक्रियाहरु | कैफियत |
| --- | --- | --- | --- |
| E1 | के तपाइको नजिकको अबिबाहित साथीले यौन सम्पर्क राखेको छ ? | 1. छ 2. छैन 3. थाहा छैन |  |
| E2 | यहा लेखिएका क्रियाकलाप मध्य तपाई कुनै पनि क्रियाकलापमा संलग्न हुनुभएको छ भने त्यस क्रियाकलापहरुलाई टिक लगाउनु होस् | 1. हस्थमैथुन 2. किस 3. योनि सेक्स 4. गुद्वार सेक्स 5. मुख मैथुन | तपाइको प्रतिक्रिया ग, घ अथवा ग र घ दुवै भए E3 मा जानुहोस् यदी ग र घ बाहेक अरु मात्र भए तपाईको सहभागिताको लागि धन्यवाद। |
| E3 | तपाइको उमेर पहिलो चोटी यौनसम्पर्क गर्दा कति थियो ? | 1. .... उमेर 2. थाहा छैन |  |
| E4 | के तपाइले पहिलो पटकको यौन सम्पर्कमा कुनै गर्भ निरोधक वा यौन रोगबाट बच्ने साधन प्रयोग गर्नु भएको थियो ? | क. गरेको थिए  ख. गरेको थिइन | प्रयोग गर्नु भएको भए E5 जानुहोस नगर्नु भएको भए E6 मा जानुहोस |
| E5 | यहा उल्लेखित मध्य कुन गर्भनिरोधक वा यौन रोगबाट बच्ने साधन प्रयोग गर्नु भएको थियो? | 1. पुरुष कण्डम 2. महिला कण्डम 3. गर्भनिरोधक चक्की(पिल्स) 4. गर्भनिरोधक सुइ(डिपो) 5. इर्मजेन्सी गर्भनिरोधक चक्की) |  |
| E6 | तपाइले गर्भनिरोधक साधन वा यौन रोगबाट बच्ने साधनको सट्टामा कुनै विकल्प जस्तै बाहिर स्खलन,सुरक्षित अबधिमा यौन सम्पर्क राख्ने जस्ता विकल्प अपनाउनु भएको थियो? | 1. अपनाएको थिए 2. अपनाएको थिइन | तपाइको प्रतिक्रिया क. भए E7 मा जानुहोस ख.भए E8 माजानुहोस |
| E7 | तपाईले कुन विकल्प अपनाउनु भयो? | 1. बाहिर स्खलन 2. सुरक्षित अबधिमा यौन सम्पर्क |  |
| E8 | तपाइको अहिले सम्म कति ओटा यौन साथी बने? | 1. ... वटा 2. याद छैन |  |
| E9 | तपाइको अन्तिम पटक यौन सम्पर्क कहिले भएको थियो? | 1. .... दिन अघि 2. ... महिना अघि |  |
| E10 | तपाइको पछिल्लो पटकको यौन साथीसंगको नाता के थियो ? | 1. श्रीमती 2. गल्फ्रेन्ड 3. साथी 4. यौनकर्मी 5. अन्य………………………. |  |
| E11 | तपाइले पछिल्लो पटकको यौन सम्पर्कमा कुनै गर्भनिरोधक वा यौन रोगबाट बच्ने साधन प्रयोग गर्नु भएको थियो ? | 1. गरेको थिए 2. गरेको थिइन | प्रयोग गर्नु भएको भए E१२ मा जानुहोस नगर्नु भएको भए E१३ मा जानुहोस |
| E12 | यहा उल्लेखित मध्य कुन गर्भनिरोधक वा यौन रोगबाट बच्ने साधन प्रयोग गर्नु भएको थियो? | क.पुरुष कण्डम  ख.महिला कण्डम  ग.गर्भनिरोधक चक्की (पिल्स)  घ.गर्भनिरोधक सुइ (डिपो)  ङ.इमार्जेन्चि गर्भनिरोधक चक्की |  |
| E13 | तपाइले गर्भनिरोधक साधन वा यौन रोगबाट बच्ने साधनको सट्टामा कुनै विकल्प जस्तै बाहिर स्खलन,सुरक्षित अबधिमा यौन सम्पर्क राख्ने जस्ता विकल्प अपनाउनु भएको थियो? | क.अपनाएको थिए  ख.अपनाएको थिइन | तपाइको प्रतिक्रिया क. भए E14 मा जानुहोस |
| E14 | तपाईले कुन विकल्प अपनाउनु भयो? | क.बाहिर स्खलन  ख.सुरक्षित अबधिमा यौन सम्पर्क |  |

**धन्यवाद**
