# Supplementary material for: Determinants of condom use during last sexual intercourse among male college youth of Kaski, Nepal: A cross-sectional survey
Source: PLoS One. 2021 Dec 30;16(12):e0261501. doi: 10.1371/journal.pone.0261501 (PMC8717988; doi:10.1371/journal.pone.0261501)
Supplement: S2 File — (DOCX) [file pone.0261501.s003.docx]

**Analysis among reported sexually active male college youth (n=903)**

| **Variables** | **Condom use during last sexual intercourse**  **Yes (634) No (269)** | | **Chi square( χ^2^)value** | **p value** |
| --- | --- | --- | --- | --- |
| **Age of the respondents** | | | | |
| 19-21 years | 405 (62.5) | 243 (37.5) | 65.2 | <0.001 |
| 22-24 years | 229 (89.8) | 26 (10.2) |  |  |
| **Level of education** | | | | |
| Undergraduate | 424 (63.6) | 243 (36.4) | 53.83 | <0.001 |
| Post graduate | 210 (89.0) | 26 (11.0) |  |  |
| **Permanent residence** | | | | |
| Pokhara | 196 (59.4) | 134(40.6) | 29.09 | <0.001 |
| Outside Pokhara | 438(76.4) | 135(23.6) |  |  |
| **Marital status** | | | | |
| Married | 79 (97.5) | 2 (2.5) | 31.75 | <0.001 |
| Unmarried | 555 (67.5) | 267 (32.5) |  |  |
| **Living arrangement** | | | | |
| With family | 252(59.6) | 171 (40.4) | 43.04 | <0.001 |
| Alone and with others ^a^ | 382 (79.6) | 98 (20.4) |  |  |
| **Knowledge about condom** (Median,3; Min-Max, 0-5) | | | | |
| Inadequate knowledge (<3) | 274 ­(90.1) | 30 (9.9) | 86.95 | <0.001 |
| Adequate knowledge (≥ 3) | 360 (60.1) | 239 (39.9) |  |  |
| **Attitude towards condom** (Median,5; Min-Max, 0-8) | | | | |
| Unfavorable attitude (<5) | 398 (92.8) | 31(7.2) | 98.93 | <0.001 |
| Favorable attitude (≥5) | 236 (49.8) | 238 (50.2) |  |  |
| **Age at sexual debut** | | | | |
| <18 years | 313 (71.3) | 126(28.7) | 0.878 | 0.367 |
| ≥18 years | 274 (68.3) | 127 (31.7) |  |  |
| **Number of sex partner** | | | | |
| One partner | 186 (47.9) | 202(52.1) | 61.35 | <0.001 |
| ≥2 partners | 448 (87.0) | 67 (13.0) |  |  |
| **Relationship with sex partner** | | | | |
| Girlfriend and wife | 566 (69.7) | 246 (30.3) | 0.986 | 0.397 |
| Others^b^ | 68 (74.7) | 23 (25.4) |  |  |

*^a^ With friends and relatives, , ^b^ Casual friend and sex worker; Statistically significant at *p<0.05*

Condom knowledge of college youths (n=903)

| **Statements*** | **In group**  **n (%)** | **% used condom** | **p value** |
| --- | --- | --- | --- |
| **1.Condoms are an effective method of preventing pregnancy** | | | |
| Agree | 556(61.6) | 49.8 | <0.001 |
| Don’t know | 4 (0.4) | 0.6 |  |
| Disagree | 343 (38) | 49.5 |  |
| **2.Condoms can be used more than once** | | | |
| Agree | 49 (5.4) | 6.9 | <0.001 |
| Don’t know | 16 (1.8) | 2.2 |  |
| Disagree | 838 (92.8) | 90.9 |  |
| **3.Condoms are an effective way of protecting against HIV/AIDS** | | | |
| Agree | 667 (73.9) | 65 | <0.001 |
| Don’t know | 17 (1.9) | 2.7 |  |
| Disagree | 219 (24.3) | 32.3 |  |
| **4.Condoms can slip off the man and disappear inside the woman's body** | | | |
| Agree | 798 (88.4) | 87.7 | 0.002 |
| Don’t know | 54 (6) | 7.6 |  |
| Disagree | 51 (5.6) | 4.7 |  |
| **5.Condoms are an effective way of protecting against sexually transmitted diseases** | | | |
| Agree | 629 (69.7) | 61.4 | <0.001 |
| Don’t know | 8 (0.9) | 1.3 |  |
| Disagree | 266 29.5) | 37.4 |  |

**Items 2, 4 are scored negatively. Items 1, 3 and 5 are scored positively*

Attitude towards condom (n=903)

| **Statements** | **In group**  **n (%)** | **% used condom** | **p value** |
| --- | --- | --- | --- |
| **1.A girl can suggest to her boyfriend that he use a condom** | | | |
| Agree | 525 (58.1) | 45.7 | <0.001 |
| Don't know | 16 (1.8) | 2.5 |  |
| Disagree | 362 (40.1) | 51.7 |  |
| **2.A boy can suggest to his girlfriend that he use a condom** | | | |
| Agree | 511 (56.6) | 53.9 | <0.001 |
| Don't know | 17 (1.9) | 2.7 |  |
| Disagree | 375 (41.5) | 43.4 |  |
| **3.Condoms are suitable for casual relationships** | | | |
| Agree | 462 (51.2) | 61.8 | <0.001 |
| Don't know | 12 (1.3) | 1.9 |  |
| Disagree | 429 (47.5) | 36.3 |  |
| **4.Condoms are suitable for steady, loving relationships** | | | |
| Agree | 364 (40.3) | 30.3 | <0.001 |
| Don't know | 12 (1.3) | 1.9 |  |
| Disagree | 527 (58.4) | 67.8 |  |
| **5.It would be too embarrassing for someone like me to buy or obtain condoms** | | | |
| Agree | 568 (62.9) | 68.8 | <0.001 |
| Don't know | 9 (1) | 0.9 |  |
| Disagree | 326 (36.1) | 30.3 |  |
| **6.If a girl suggested using condom to her partner, it would mean that she didn't trust him** | | | |
| Agree | 463 (51.3) | 32.6 | <0.001 |
| Don't know | 16 (1.8) | 2.1 |  |
| Disagree | 424 (47) | 65.3 |  |
| **7.Condoms reduce sexual pleasure** | | | |
| Agree | 677(75) | 78.9 | <0.001 |
| Don't know | 22 (2.4) | 3 |  |
| Disagree | 204(22.6) | 18.1 |  |
| **8.If unmarried couples want to have sexual intercourse before marriage, they should use condoms** | | | |
| Agree | 477 (52.8) | 58.4 | <0.001 |
| Don't know | 16(1.8) | 2.5 |  |
| Disagree | 410 (45.4) | 39.1 |  |
