# Supplementary material for: Determinants of condom use during last sexual intercourse among male college youth of Kaski, Nepal: A cross-sectional survey
Source: PLoS One. 2021 Dec 30;16(12):e0261501. doi: 10.1371/journal.pone.0261501 (PMC8717988; doi:10.1371/journal.pone.0261501)
Supplement: S3 File — (DOCX) [file pone.0261501.s004.docx]

**1. Knowledge scale**

The Cronbach alpha of knowledge scale is 0.56

| **Variable** | **Cronbach alpha** | **Number of items** |
| --- | --- | --- |
| Knowledge | 0.56 | 5 |

**Summary Item Statistics of knowledge scale**

The mean covariance between the items is 0.08 and the item mean variance was 1.95.

|  | **Mean** | **Minimum** | **Maximum** | **Range** | **Maximum / Minimum** | **N of Items** |
| --- | --- | --- | --- | --- | --- | --- |
| Item Means | 1.957 | 1.291 | 2.877 | 1.586 | 2.229 | 5 |
| Item Variances | .442 | .221 | .680 | .460 | 3.083 | 5 |
| Inter-Item Covariances | .089 | -.059 | .413 | .472 | -6.965 | 5 |
| Inter-Item Correlations | .132 | -.175 | .703 | .878 | -4.016 | 5 |

**Principle component analysis**

For construct validity, principal component analysis was conducted which revealed one factor with eigenvalues greater than 1 and the total variance explained was 47.31.

| **Total Variance Explained** | | | | | | |
| --- | --- | --- | --- | --- | --- | --- |
| Component | Initial Eigenvalues | | | Extraction Sums of Squared Loadings | | |
|  | Total | % of Variance | Cumulative % | Total | % of Variance | Cumulative % |
| 1 | 2.366 | 47.319 | 47.319 | 2.366 | 47.319 | 47.319 |
| 2 | .992 | 19.844 | 67.164 |  |  |  |
| 3 | .951 | 19.016 | 86.180 |  |  |  |
| 4 | .413 | 8.268 | 94.448 |  |  |  |
| 5 | .278 | 5.552 | 100.000 |  |  |  |
| *Extraction Method: Principal Component Analysis*. | | | | | | |

**2.Attitude scale**

The Cronbach alpha of attitude scale is 0.73

| **Variable** | **Cronbach alpha** | **Number of items** |
| --- | --- | --- |
| Attitude | 0.73 | 8 |

**Summary Item Statistics of attitude scale**

The mean covariance between the items is 0.18 and the item mean variance is 1.50.

|  | **Mean** | **Minimum** | **Maximum** | **Range** | **Variance** | **N of Items** |
| --- | --- | --- | --- | --- | --- | --- |
| Item Means | 1.509 | 1.267 | 1.737 | .470 | .019 | 8 |
| Item Variances | .715 | .449 | .857 | .408 | .015 | 8 |
| Inter-Item Covariances | .183 | -.189 | .674 | .863 | .090 | 8 |
| Inter-Item Correlations | .245 | -.244 | .955 | 1.199 | .163 | 8 |

**Principal component analysis**

Principal component analysis for assessing construct validity revealed two factor with eigenvalues greater than 1 and the total variance explained was 49.32 and 23.96 respectively.

| Component | Initial Eigenvalues | | | Extraction Sums of Squared Loadings | | |
| --- | --- | --- | --- | --- | --- | --- |
|  | Total | % of Variance | Cumulative % | Total | % of Variance | Cumulative % |
| 1 | 3.946 | 49.322 | 49.322 | 3.946 | 49.322 | 49.322 |
| 2 | 1.917 | 23.964 | 73.286 | 1.917 | 23.964 | 73.286 |
| 3 | .723 | 9.039 | 82.325 |  |  |  |
| 4 | .532 | 6.654 | 88.979 |  |  |  |
| 5 | .371 | 4.634 | 93.613 |  |  |  |
| 6 | .296 | 3.696 | 97.309 |  |  |  |
| 7 | .171 | 2.139 | 99.448 |  |  |  |
| 8 | .044 | .552 | 100.000 |  |  |  |
