# Supplementary material for: Determinants of condom use during last sexual intercourse among male college youth of Kaski, Nepal: A cross-sectional survey
Source: PLoS One. 2021 Dec 30;16(12):e0261501. doi: 10.1371/journal.pone.0261501 (PMC8717988; doi:10.1371/journal.pone.0261501)
Supplement: S4 File — (DOCX) [file pone.0261501.s005.docx]

Table. Weighted and Unweighted number of students in different levels

| Level (A) | Number of classes(B) | Total male students (C) | Weightage (proportion)  (D)=Ci/N | Weighted number (E)=Dn | Unweighted number (sample) (F) | Unweighted proportion  (G)=(Fi/E)*D | Chi-square statistic, df (p-value) |
| --- | --- | --- | --- | --- | --- | --- | --- |
| Bachelor First Year | 87 | 1376 | 0.2161 | 78 | 72 | 0.1995 | 0.60, 4 (.96) |
| Bachelor Second Year | 86 | 1041 | 0.1634 | 59 | 62 | 0.1717 |  |
| Bachelor Third Year | 84 | 1023 | 0.1606 | 58 | 54 | 0.1495 |  |
| Bachelor Fourth Year | 58 | 935 | 0.1468 | 53 | 55 | 0.1523 |  |
| Master First Year | 21 | 1994 | 0.3130 | 113 | 118 | 0.3269 |  |
| Total |  | N=6369 | 1.00 | n=361 | n=361 | 0.99 |  |
