# Supplementary material for: Determinants of condom use during last sexual intercourse among male college youth of Kaski, Nepal: A cross-sectional survey
Source: PLoS One. 2021 Dec 30;16(12):e0261501. doi: 10.1371/journal.pone.0261501 (PMC8717988; doi:10.1371/journal.pone.0261501)
Supplement: Attachment — Submitted filename: New comments and justifications.docx [file pone.0261501.s006.docx]

**1. Did the authors present any new data in this submission that were not previously presented in the published JHAS article?**

Yes, there are new data in the submission that were not previously presented in the published JHAS article. Some variables as background and behavioral variables are similar.to the JHAS but the new data are of condom knowledge (its items, categories, item wise association of knowledge with condom use ), condom attitude (items , categories, item wise association of attitude with condom use ), Condom use during last sexual intercourse, Alternative measures used by condom non-users (Safe period, withdrawal, contraceptive pills, contraceptive injection, emergency contraceptive pills used by respondents or his partner) , examination of the strength of association between condom use during last sexual intercourse and related variable through model construction.

**2. Did the authors perform any additional experiments or collect any additional data that were not a part of the study from the published JHAS article?**

The JHAS article was mainly focused for revealing the sexual behavior of the youths which mainly focused on nature of sexual activities that youth performed as how many performed masturbation, kissing, vaginal, anal and oral sex in their life prior to the study .The dependent variable for that study was having sexual intercourse till date of survey or not. So the analysis was considered by taking dependent variable as whether youths performed sexual intercourse or not with the various independent variables of the study as: background and behavioral variables.

The present study is completely different from the previous. It is in a sense that the dependent variable of this study is condom use among youths which was only assessed from the youths who were sexually active before six months of survey. Similarly additional data collected were :condom knowledge, attitude towards condom, whether youths used condom during their last sexual intercourse or not, did condom non users/their partners used any alternative like safe period, withdrawal, contraceptive pills, contraceptive injection, emergency contraceptive pills.

The analysis is completely different between this two studies as this study which is submitted in PLOS performed analysis as construction of models, conduction of normality test ,multicollinearity test, item wise association of knowledge and attitude with condom use, descriptive analysis of alternative measures of condom use .Similarly for validation of scale also various statistical analyses were performed.

**Rebuttal of review**

We have incorporated all of your suggestions in the revision except the weighted calculations. We calculated the weighted estimator of the sample and found 1.04 when the formula W_i_= K/Si (Tyagi, S., Sharma, B., Singh, P. and Dobhal, R., 2013. Water quality assessment in terms of water quality index. american Journal of water resources, 1(3), pp.34-38.) was applied. Since the estimator is minimal, it does not make a large effect, and therefore need not be carried further calculations out as implicitly mentioned by Korn and Graubard (Korn, E.L. and Graubard, B.I., 1995. Examples of differing weighted and unweighted estimates from a sample survey. The American Statistician, 49(3), pp.291-295).
