# Supplementary material for: Determinants of condom use during last sexual intercourse among male college youth of Kaski, Nepal: A cross-sectional survey
Source: PLoS One. 2021 Dec 30;16(12):e0261501. doi: 10.1371/journal.pone.0261501 (PMC8717988; doi:10.1371/journal.pone.0261501)
Supplement: Attachment — Submitted filename: Response to reviewers_Jul26-2021.docx [file pone.0261501.s007.docx]

26^th^ July, 2021

Dear editor/reviewers,

PLOS ONE

**Sub: Response to Editors and Reviewers**

We are very much thankful to the editor and the reviewers for your generous comments on the manuscript entitled " Determinants of condom use during last sexual intercourse among male college youths of Kaski, Nepal: A cross-sectional survey". We hope the scientific comments you aroused have helped rectify the manuscript and accordingly, we have revised it by addressing all the concerns raised by editor and reviewers. The responses along with the comments are described in this rebuttal letter. We also apologize for a bit delayed response.

**Editor comments and changes /justification**

1. **Comment # 1:** A first required change is the use of “male college youths” in some parts of the text instead of “male college youth”, which is also used. Youth can be plural, and use of “youths” should be avoided. Please replace all uses of “youths” for “youth”.

**Our response:** We thank the editor for this lexical error and all "youths” are now replaced by “youth” in the manuscript.

2. **Comment # 2:** Among the various health issues related to sexual and reproductive health, the major concern of youth is that they expose to the Sexually Transmitted Infections”. Please rephrase. The sentence does not make grammatical sense. Who is “they”?.Also, use “a major concern”, it is unclear that it is “the” major concern, and that does not stem from the reference given.

**Our Response:** Thank you for this lexical and grammatical issue. We have, now, rephrased it as: “The youth face various health problems that can affect their quality of life. First and foremost is HIV/AIDS, which is increasingly affecting young people.” We hope the sentence is sensible now.

3. **Comment # 3:** But the common problem faced by most of the countries is that young people are reaching puberty earlier and are often engaged in sexual activities”. Countries do not face problems, and it is debatable whether reaching puberty earlier and engaging in sexual activities is a problem. You have to use neutral language. The same for the following sentence “. You could write something as “A common pattern is for youth reaching puberty and engaging in sexual activity earlier.

**Our response:** Thank you again for notifying us. The sentence "But the common problem faced by most of the countries is that young people are reaching puberty earlier and are often engaged in sexual activities" is now written as "A common pattern is for youth reaching puberty and engaging in sexual activity earlier".

4. **Comment # 4:** Evidence shows earlier sexual initiation to be associated with having multiple sex partners and higher risk of sexually transmited infections”. You should introduce at this stage evidence from the prior study in JHAS such as proportion ever sexually active and multiple partnership in Kaski compared to DHS or the other studies in Achham and Kathmandu.

**Our response:** We are thankful to the generous comment. Prior study of JHAS ,Achham ,Kathmandu and DHS data are now added and arranged with this sentence. We found it as improving the rigor of the manuscript.

5. **Comment # 5:** These existing literature of Nepal indicates that the sexual behavior of youths of Nepal are risky.” Revise. It does not make grammatical sense.

**Our response:** The sentence is now revised as " Thus, the literature suggests that the sexual activity of youth in Nepal is risky."

6. **Comment # 6:** You also need to refer to the other article in the statement of the research objectives. You just say “Despite the efforts from various level, the prevalence of condom use among youths is low [12]. So this study examined the condom use at last sexual intercourse among the male college youths.” You are not looking at this in the vacuum. You are looking at youth in a particular city where you have studied sexual behaviour. This is important as commented before.

**Our response:** We are thankful for the comments. More literatures for the justification have been added in the revised manuscript

7. **Comment # 7:** If a submitted study replicates or is very similar to previous work, authors must provide a sound scientific rationale for the submitted work and clearly reference and discuss the existing literature. Submissions that replicate or are derivative of existing work will likely be rejected if authors do not provide adequate justification (<http://www.plosone.org/static/publication#results>). The mentioned paper was not included among the reference or put in context as required, and if the required justification and incorporation in the study is not given that is grounds for rejection”. This justification needs to be incorporated in the text as an antecedent of the current study. Also you are saying in the response that this study uses “new data”. You have to clarify that: Was there one interview which is common to both studies or two different interviews? This is confusing.

**Our response:** In line with the editor’s comment, we have now introduced our prior study of JHAS in the sentence "Evidence shows earlier sexual initiation to be associated with having multiple sex partners  and higher risk of sexually transmitted infections” ,and the mentioned paper is also included in the reference in the 8^th^ number. In addition, the mentioned paper study is also discussed in the context of multiple sex partners at the discussion section.

- It is also explained as the rationale in the last paragraph of introduction that "Previous study conducted among male college youth of Kaski district revealed that the substantial proportion of male college youth was engaged in risky sexual behavior where more than half (51.61%) of the male youth had not used condom at an every act of sexual intercourse with commercial sex worker. So this study is important to examine the prevalence and determinants of condom use at last sexual intercourse among male college youth of Kaski district"
- Regarding the interview, two different interviews were conducted. Prior study (published in JHAS) among the participants who were sexually active during the last one year period, after preliminary findings of which further intrigued us to think about the condom use during the last vaginal/anal intercourse (within 6 months) would be even more interesting and important from the scientific point of view, and so accessed among those and included as per the criteria, which helped us to get to 361. This study is, so, the further culmination of previous study which, probably, was able to answer, another but an important research question.

8. **Comment # 8:** It is evident that several factors influence the condom use among youth as their sociodemographic characteristics: residence, education, living arrangement”. Nothing is evident. That is not, in particular, evident. Also, verb and noun should agree in number. The English language of the article really needs improvement.

**Our response:** It is revised as "Studies have documented that living with parents and higher education level are the important predictors of consistent condom use"

9. **Comment # 9:** Sample weights are still not used. The description of sampling has been provided: “The total number of classes of undergraduate level as: Bachelor first ,second, third and fourth and year was 87,86.84 and 58 respectively and of post graduate was 21.As per the population proportion to size the total number of classes to be taken from undergraduate to post graduate level was 46 .Thus the 46 classes were selected by generating random number and all male college youths of the selected classes were the study population.”. First, language should still be improved. Second, it emerges that there are unequal probabilities of selection. Students in smaller classes have a higher probability of selection since there is a random sample of classes, not of students. The argument given for not using weights is a reference to an article on water management that is not relevant methodologically, and the reproduced equation does not make sense in this setting. You are not calculating a weighted average. You are estimating a GLM model with unequal probabilities of selection. This requires use of normalized weights with an average level of one.

**Further commented/clarification on 16^th^ July, 2021 (upon our request):** Regarding the question of Dr. Adhikari, please reply to him that I'd be glad to see their resubmission. Turning to their question regarding point 9:

Upon re-reading of the sampling procedure, it seems that there might be (prior) equal probabilities of selection since all male students in each sampled class were targeted so the probability of selection is given by the proportion of classes surveyed to all classes. There would be unequal probabilities if a fixed number of students were sampled, but this is not apparently the case. However, ex-post there are still different probabilities of sampling in each of the education levels. Eg: What if all the 46 randomly selected classes belonged to the 87 first-year classes? The prior probability of choosing students from each level is the same as in the population, but, after the sample is taken, the proportion of students from each level in the sample could be as drastic as only first-year students. Note you could use weights to adjust for this (unless one education level is totally missing!) defined as the proportion of level i (eg: first year students) in the population divided by the proportion of first year students in your sample. In this way you give more weight to students from levels that are underrepresented and the weights are normalized (have an average of one). This is equivalent to using strata. You can use these weights (defined at the student-year level, first to fourth and postgraduate) in your analysis. Alternatively, you can show that your sample is balanced, (eg, if all weights calculated in this way are very close to one) and not use weights.

Still, the paragraph was hard to read. Eg: "and all male college youths of the selected classes were the study population". This confuses sample and population. All the male college youths in the selected classes constitute your sample. The population includes all male college youths, in all classes irrespective of sampling status.

Second, the sentence "As per the population proportion to size the total number of classes to be taken from undergraduate to post graduate level was 46" is also confusing". Do you mean something like "based on average classroom size, 46 classes would have to be targeted to reach desired sample size". If this is so, then put the paragraph that follows on targeted sample size before and not after describing the sample selection. Note that given unequal classroom sizes what varies depending on the classroom sample is sample size, which is not fixed.

In short: there is a need for a thorough language check. Regarding weights, you can either show that your sample is balanced and the proportions in the sample of students by year are similar to the proportions in the population, or else use weights as suggested (ratio of the previously mentioned proportions).

**Our response:** We are very much thankful to your comments and the language feedback. **Firstly,** regarding the thorough language check, we took it seriously, and all three authors, checked and peer reviewed the language after corrected in *online Grammerly*. that was checked after revising the manuscript as commented and feedback. The illogical, insensible and unintelligible sentences that have been commented have been revised in consultation with the professional language expert.

**Secondly,** and may be, even more importantly, in response to the weightage calculation/balanced sample, we are grateful to the reviewers and editors, for providing the academic suggestions. As per your suggestions and to be more assured, we consulted the biostatistician and his verdict was consistent with your later one. Henceforth, we calculated the weighted proportion of the actual male youth and compared it with the unweighted number that was sampled for study, and it revealed that the sample is balanced. We also carried out the chi-square test if there exists any association between the number of students of weighted and unweighted and the finding was insignificant (chi-square statistic, 0.60; p-value, 0.96) and concluding that there was no association between the two data sets (supporting file 5).

**Thirdly,** the doubt that has been aroused by the reviewer that the disproportionate sampling would have occur and all the classrooms would have been selected only from bachelor level, we were conscious about that, and we had then selected the classrooms from each of bachelor’s 1^st^, 2^nd^, 3^rd^, 4^th^, and master’s 1^st^ years in accordance with the proportion to the number of students in the classrooms. So, the representation from all the levels was assured (supporting file 5).

10. **Comment # 10:** The section on the results of the multivariate analysis reads very poorly. It should be a little more than reproducing the elements of the table. Think of the reader.

**Our response:** Thinking the reader, as per your suggestions, the manuscript in now revised and added the explanation of each model so that author can connect the interpretation with table easily.

11. **Comment # 11:** Discussion: Needs improvement in the language and the substance. For instance, what does it mean “The study revealed the aspects of youth's sexual behavior that may point to advance the well-being of youth's reproductive and sexual health”.Besides the clumsy redaction that does not seem to be the purpose of the article. You have looked at the determinants of condom use at last intercourse, and there are no policy variables included.

**Our response:** “The study revealed the aspects of youth's sexual behavior that may point to advance the well-being of youth's reproductive and sexual health” is replaced by The study examined the determinants of condom use during last sexual intercourse among sexually active male youth of Kaski, Nepal. We have also thoroughly checked the manuscript, that has already clarified in response of comment 9.

As the study had focused on determinants of condom use during last sexual intercourse and had not included policy variables so policy related aspects are no more discussed now in the revised manuscript and is omitted.

12. Upon re-reading of the sampling procedure, it seems that there might be (prior) equal probabilities of selection since all male students in each sampled class were targeted so the probability of selection is given by the proportion of classes surveyed to all classes. There would be unequal probabilities if a fixed number of students were sampled, but this is not apparently the case. However, ex-post there are still different probabilities of sampling in each of the education levels. Eg: What if all the 46 randomly selected classes belonged to the 87 first-year classes? The prior probability of choosing students from each level is the same as in the population, but, after the sample is taken, the proportion of students from each level in the sample could be as drastic as only first-year students. Note you could use weights to adjust for this (unless one education level is totally missing!) defined as the proportion of level i (eg: first year students) in the population divided by the proportion of first year students in your sample. In this way you give more weight to students from levels that are underrepresented and the weights are normalized (have an average of one). This is equivalent to using strata. You can use these weights (defined at the student-year level, first to fourth and postgraduate) in your analysis. Alternatively, you can show that your sample is balanced, (eg, if all weights calculated in this way are very close to one) and not use weights.

**Our response:** This comment was given on 16^th^ Jul, 2021 and included in comment 9, above, and responded accordingly.

**Comments of Reviewer 1 and changes/justification**

1. Pokhara and Kaski are interchangeably used in the manuscript, such as in the last sentence in the introduction and some parts of the results. Do both names refer to the same district?

**Our response:** We are thankful to your comments. Consistency has been maintained through out the manuscript by using the word Kaski.

2. In the sub-section of questionnaire, please provide the citations from where questionnaires were adopted.

**Our response**: Dear reviewer, citations are now provided while explaining the questionnaires.

3. In the methods section, please make clear whether any records with missing data that were removed from 361 males who engaged in sexual intercourse in last 6 months.

**Our response:** Dear reviewer, it is now explained in the statistical method section of manuscript and written as Altogether there were 74 ambiguous responses of the sexually active male youth in which 12 responses were of sexually active male youth within six months preceding the survey. Likewise, among 21 incomplete responses, 6 largely incomplete responses were of sexually active male youth within six months preceding the survey. Thus, ambiguous responses that were logically inconsistent were excluded after verification through the consultation of reproductive health experts and incomplete data were also eliminated from the analysis.

4. For bivariate analysis (Table 6), please mention the reference category or group for all variables. No reference group was mentioned for the age of the respondent and educational level.

**Our response:** The reference group is now mentioned for the age of the respondent and educational level.

5. For the same table, please check again the association between the number of sex partners and condom use. From the table, those who had 2 partners or more were more likely to use a condom at last sex (OR=8.91; 95%CI=5.21-15.24). Surprisingly, based on the differences in the proportion of condom use by the number of sex partners, the proportion of condom use was lower among those with ≥ 2 partners (11.01%) compared to those with 1 partner only (52.44%). Please check again the results whether there was a mistake in coding the categories. Please check also for other variables: level of education, relationship with sex partners, and provide interpretation for Table 6.

**Our response:** The result of all variables of Table 6 was checked and it was found that there was mistake on data for the association between the number of sex partners and condom use. The proportion of condom users among ≥ 2 partners is 194 (89 %) and with 1 partner only is 68(47.5%).For other variable also it was checked and no mistake was found.

6. Authors reported a high correlation between the age of the respondents and level of education (r=0.9) and decided to not include both variables in the multivariate model. However, two highly correlated variables indicate that both cannot be included in the multivariate model in a time, but we can select one of them. Therefore, authors can include either age of the respondents or educational level in the model. I suspect that educational level can be strongly associated with condom use.

**Our response:** Educational level is now added in the model.

7. For multivariate analysis (Table 7), I am wondering why the number of sex partners was not statistically significant associated with condom use. The 95%CI (3.01 – 11.07) informs that the value of one (1) is not within the intervals. Please re-check the results.

**Our response**: The result was checked and it was found that sex partners was statistically significant with condom use in table 7 so the manuscript is revised as per it.
